# Racial and ethnic differences in tumor characteristics among endometrial cancer patients in an equal-access healthcare population

**DOI:** 10.1007/s10552-023-01716-9

**Published:** 2023-07-12

**Authors:** Daniel Desmond, Zhaohui Arter, Jeffrey L. Berenberg, Jeffrey L. Killeen, Kristen Bunch, Melissa A. Merritt

**Affiliations:** 1 Walter Reed National Military Medical Center, 4494 Palmer Rd N, Bethesda, MD 20814, 301-319-4627 USA; 2 University of California, Irvine Medical Centeur, CA Irvine, USA; 3 Tripler Army Medical Center, Honolulu, HI USA; 4 University of Hawaii Cancer Center, Honolulu, HI USA; 5 Department of Pathology, John A. Burns School of Medicine, University of Hawaii, Honolulu, HI USA; 6 Kapiolani Medical Center for Women and Children, Honolulu, HI USA; 7 The Daffodil Centre, The University of Sydney, a joint venture with Cancer Council NSW, Sydney, NSW Australia

**Keywords:** Endometrial cancer, Endometrioid, Ethnicity, Race, Military

## Abstract

**Purpose:**

There are racial and ethnic differences in endometrial cancer incidence and mortality rates; compared with Non-Hispanic White women, Black women have a similar incidence rate for endometrial cancer, but their mortality is higher. Pacific Islander women may also have worse outcomes compared to their White counterparts. We assessed tumor characteristics and adjuvant therapy by racial and ethnic group among endometrial cancer patients treated within the Military Health System, an equal access healthcare organization.

**Methods:**

We retrospectively identified women diagnosed with invasive endometrial cancer among US Department of Defense beneficiaries reported in the Automated Central Tumor Registry database (year of diagnosis: 2001–2018). We compared tumor characteristics and receipt of adjuvant therapy across racial and ethnic groups using Chi-square or Fisher tests. Hazard ratios (HRs) and 95% confidence intervals (CIs) for risk of all cause mortality were calculated using Cox proportional hazards regression models adjusting for age at diagnosis, adjuvant therapy, histology and stage.

**Results:**

The study included 2574 endometrial cancer patients [1729 Non-Hispanic White, 318 Asian, 286 Black, 140 Pacific Islander and 101 Hispanic women]. Among all cases, a higher proportion of Black patients had non-endometrioid histology (46.5% versus ≤ 29.3% in other groups, P < 0.01) and grade 3–4 tumors (40.1% versus ≤ 29.3% in other groups, P < 0.01). In multivariable Cox models, compared with Non-Hispanic White cases, Black endometrial cancer patients had a higher mortality risk (HR 1.43, 95% CI, 1.13–1.83). There was no difference in mortality risk for other racial and ethnic groups.

**Conclusion:**

Black patients with endometrial cancer presented with more aggressive tumor features and they had worse overall survival compared with patients in other racial and ethnic groups. Further study is needed to better direct preventive and therapeutic efforts in order to correct endometrial cancer disparities in the future.

**Supplementary Information:**

The online version contains supplementary material available at 10.1007/s10552-023-01716-9.

## Introduction

Endometrial cancer is the most common gynecologic malignancy in the western world and the fourth most common cancer overall in women in the United States [1]. Fortunately, 67% of patients present with localized disease which is associated with a 5-year relative survival rate of 95% [2]. However, unlike most cancers, the incidence and mortality of endometrial cancer has been rising [1]. Women with early menarche, late menopause, anovulation, unopposed estrogen use and obesity are at increased risk to develop endometrial cancer [3]. The obesity epidemic has been suggested as a possible driver of the rising incidence of endometrial cancer in the United States [4]. Obesity has a more pronounced association with the more common and less aggressive endometrioid endometrial cancer (EEC) which represents about 85% of cases [5]. Conversely, non-endometrioid endometrial cancer (non-EEC), is classically a more aggressive disease. Non-EEC, which disproportionately affects Non-Hispanic Black (hereafter referred to as Black) women [6], is increasing in incidence and has been suggested as the primary reason for the 1.9% annual increase in endometrial cancer mortality [1, 6]. Although Black women have similar age-adjusted incidence rates of endometrial cancer as their Non-Hispanic White (hereafter referred to as White) counterparts [6, 7], they have an 80% increased risk for mortality regardless of stage or histology [8]. There is ongoing research into the causes of this disparity including inquiries into age at time of presentation, sociodemographic characteristics, variability in somatic mutational pattern and standard of care delivery [9–12].

Two retrospective analyses of data from Surveillance Epidemiology and End Results (SEER) and the National Cancer Database (NCD) reviewed adherence to National Comprehensive Cancer Network guidelines and established standards of care, respectively, and found that Black patients and those of low socioeconomic status (regardless of race or ethnicity) were less likely to receive standard of care treatment when compared with White patients or those of higher socioeconomic status [8, 10]. Adjustment for perfect adherence to established standards of quality care in patients with stage III disease did not remove a statistically significant higher risk for all-cause mortality in Black versus White endometrial cancer patients [5-year overall survival (OS): HR 1.35, 95% confidence interval (CI), 1.22–1.50]. It is unknown what drives this disparity in outcomes but differences in access to care have been suggested as a possible cause [4].

The United States Department of Defense (DoD) military health system provides equal access to care for all beneficiaries regardless of race and ethnicity or socioeconomic background. The Automated Central Tumor Registry (ACTUR) was established in 1986 as the cancer registry system for the DoD. Previous studies using ACTUR data have shown a higher proportion of non-endometrioid histology in Black versus White endometrial cancer patients (48 versus 24%) and increased all-cause mortality in Black versus White patients (HR: 1.64, 95% CI = 1.19 to 2.27, adjusted for age at diagnosis, diagnosis period, tumor stage, histology, and receipt of adjuvant treatment) [13, 14]. The current study represents the largest ACTUR endometrial cancer study to date with the most contemporary study population and extends analysis to include additional racial and ethnic groups (i.e., Asian, Pacific Islander and Hispanic patients).

## Methods

### Study population

This study was conducted in patients of the DoD military health system who receive their medical care free of charge. Of the 9.6 million beneficiaries of the military health system, nearly half of them (active duty/reserve and family members) are provided allowances for housing and subsistence that are related to rank and time in service. There are equal opportunities for career advancement and educational opportunities for those who serve in the military. The authors of this paper understand that there are complex structural barriers to healthcare access that persist regardless of equal pay, housing and provider availability; however, the benefit of improved access to care on cancer-related outcomes has been demonstrated in several tumor types in patients treated within the military health system [15–17].

Data from the DoD ACTUR database were used to conduct this study. Briefly, the ACTUR database includes data on DoD beneficiaries (including active-duty military personnel, retirees and their dependents) who are diagnosed with cancer or receive cancer treatment at military facilities [13]. Local tumor registrars abstract and enter data for newly diagnosed cancer patients in consultation with gynecologic oncologists. Endometrial cancer was defined as International Classification of Diseases for Oncology 2nd or 3rd revision codes C54.0-54.9 and C55.9 (further details and exclusions are shown in Supplementary Table 1). This study included invasive endometrial cancer cases diagnosed between 2001 and 2018. Data were abstracted from the ACTUR in February 2021 on tumor histology, stage [local, regional, distant or unknown by combining Surveillance, Epidemiology and End Results (SEER) summary stage variables], grade [well (G1), moderate (G2), poor (G3), undifferentiated (G4) or unknown], information on the first course of treatment [surgery (yes/no); receipt of adjuvant chemotherapy and/or radiation] and vital status (all-cause mortality). Racial and ethnic groups were recorded in the registry database using: (1) information that is documented in the beneficiary medical record [which includes health data from the DoD, Department of Veterans Affairs and private sector partners) and outside/community records or documents]; (2) observations made by the treating/managing physicians; or (3) information obtained directly from the patient’s response to the Oncology/Registry Patient Questionnaire (when applicable).

We identified *N* = 2966 endometrial cancer cases and the following exclusions were applied: not invasive (*N* = 20); non-epithelial (*N* = 153); and cases that were not included in the five major racial and ethnic groups that were the focus of this study (*N* = 219) leaving 2574 cases for the study. In addition, we conducted survival analyses focusing on all cause mortality as the outcome. Death due to endometrial cancer could not be examined because data on the cause of death were not available for most patients. Additional exclusions were applied for the survival analysis: unknown vital status (N = 5); date of diagnosis was the same as the date of death (N = 2); no surgery (N = 157) or missing surgery (N = 31); missing stage (N = 61) leaving 2318 cases for survival analysis. This study was approved by institutional review boards at all participating institutions.

### Statistical analysis

We evaluated whether there were differences in tumor characteristics and receipt of the first course of adjuvant therapy by racial and ethnic group using the Chi-square test or Fisher test for small sample sizes. We used Cox proportional hazards regression models to calculate hazard ratios (HRs) and 95% confidence intervals (CIs) for the risk of all cause mortality across racial and ethnic groups. Person-time was calculated as the number of days between a patient’s date of diagnosis until the date of last contact or censoring, whichever occurred first. Multivariable models were adjusted for covariates selected a priori because of their known influence on risk of mortality: age at diagnosis (continuous); and receipt of the first course of adjuvant therapy [no adjuvant therapy (reference), radiation therapy, chemotherapy, radiation and chemotherapy, missing]. Multivariable models were stratified by: histology [EEC, non-EEC]; and stage [localized, regional, distant]. The proportional hazards assumption was tested using the method described by Grambsch and Therneau [18]. Histology and stage violated proportional hazards therefore these variables were included as strata terms in the model. No violation of proportional hazards was observed for age and adjuvant therapy.

Receipt of adjuvant therapy (receipt of radiotherapy only, chemotherapy only or both) was taken as an indicator for the presence of adverse tumor characteristics that were not available in the current dataset, including invasion of for example the lymphovascular space, tumor size and myometrial invasion [19]; this has particular relevance for patient subgroups (e.g., low grade EEC) who would not typically receive adjuvant therapy. We classified cases according to their age at diagnosis (< 50 years and 50 + years) to approximate premenopausal and postmenopausal disease, respectively, similar to an earlier report [13]. There may be clinical, pathological and etiologic differences for women who develop premenopausal endometrial cancer; this concept has been explored in breast cancer [20] but not as we are aware for endometrial cancer. Descriptive analyses were age-standardized [21]. All statistical tests were two-sided and *P*-values < 0.05 were considered statistically significant. Statistical analyses were performed using R version 4.1.0 [22].

In accordance with the journal’s guidelines, we will provide our data for independent analysis by a selected team for the purposes of additional data analysis or for the reproducibility of this study in other centers if such is requested.

## Results

When comparing age-standardized characteristics among endometrial cancer patients by racial and ethnic group, the percentage of fatal cases (death from all causes) was highest among Black women (33.1% followed by 29.4% in Hispanic patients, and ≤ 27.9% in other racial and ethnic groups) (Table 1). Endometrioid histology was most common in all racial and ethnic groups (range, 56.0% of Black patients to 74.7% of White patients) and Black patients had a higher proportion of serous histology (13.9% serous versus ≤ 5.5% in other racial and ethnic groups). The mean age at diagnosis and receipt of surgery was similar across racial and ethnic groups.

**Table 1:** Age standardized characteristics of invasive endometrial cancer cases by race/ethnicity in the ACTUR database

	Overall (n = 2574)	Non-Hispanic White (n = 1729)	Black (n = 286)	Asian (n = 318)	Pacific Islander (n = 140)	Hispanic (n = 101)
Fatal (all causes), %^a^	27.7	27.9	33.1	23.9	17.8	29.4
Age at diagnosis (years)^b^	58.9 (11.4)	59.3 (11.4)	60.9 (11.9)	57.4 (10.2)	55.4 (11.0)	56.4 (12.7)
**Histology**						
Endometrioid, %	71.9	74.7	56.0	74.1	70.9	70.6
Adenocarcinoma NOS, %	12.1	12.2	14.6	7.7	13.2	17.3
Serous, %	5.0	3.3	13.9	4.8	5.5	4.9
Other histology, %	11.0	9.7	15.5	13.5	10.4	7.2
**First course treatment** ^c^						
Surgery: yes, %	93.8	94.7	93.7	91.1	88.5	94.7
Surgery: no, %	6.2	5.3	6.3	8.9	11.5	5.3

Values are means(SD) for continuous variables; percentages for categorical variables, and are standardized to the age distribution of the study population

^a^ Data on vital status were missing for 0.2% of cases

^b^ Value is not age adjusted

^c^ Data on surgery were missing for 1.3% of cases

Abbreviations:

NOS, not otherwise specified

When comparing tumor characteristics and receipt of adjuvant therapy (an indicator of adverse tumor characteristics) across racial and ethnic groups, we observed significant differences for histology, stage and grade among all cases (P ≤ 0.04) and among cases who were age 50 + years at diagnosis (P ≤ 0.01) (Table 2). A higher percentage of Black patients had more aggressive tumor characteristics compared with most other racial and ethnic groups. For example, among cases who were age 50 + years at diagnosis, Black patients had a higher proportion of non-EEC histology (48.0% of Black patients versus ≤ 30.1% among other groups, P < 0.01) and grade 3–4 disease (41.2% of Black patients compared with ≤ 32.7% among other groups, P < 0.01). A higher percentage of Black and Asian patients were diagnosed with regional/distant stage disease (age 50 + years, 32.3% of Black, 32.7% of Asian patients compared with ≤ 28.8% for other racial and ethnic groups, P = 0.01). In contrast, in younger patients (age < 50 years) only grade differed by racial and ethnic group (grade 3–4 disease was observed in 33.3% of Black patients compared with ≤ 21.7% patients in other groups, P < 0.01).

**Table 2:** Endometrial cancer patient tumor characteristics and adjuvant treatment by racial and ethnic group and age

Age at diagnosis	Tumor/ treatment variable	Values	Non-Hispanic White	Black	Asian	Pacific Islander	Hispanic	p.value
All	Histology	Endometrioid	1290 (74.6%)	153 (53.5%)	236 (74.2%)	99 (70.7%)	73 (72.3%)	< 0.01
		Non-endometrioid	439 (25.4%)	**133 (46.5%)**	82 (25.8%)	41 (29.3%)	28 (27.7%)	
	Stage	Local	1255 (76.1%)	189 (70.3%)	211 (69%)	99 (76.7%)	73 (73.7%)	0.04
		Regional or Distant	394 (23.9%)	**80** **(29.7%)**	**95** **(31.0%)**	30 (23.3%)	26 (26.3%)	
	Grade	1–2	1125 (83.1%)	118 (59.9%)	201 (77.6%)	70 (70.7%)	55 (71.4%)	< 0.01
		3–4	229 (16.9%)	**79** **(40.1%)**	58 (22.4%)	29 (29.3%)	22 (28.6%)	
< 50 years	Histology	Endometrioid	219 (78.2%)	23 (63.9%)	47 (77%)	25 (69.4%)	22 (78.6%)	0.33
		Non-endometrioid	61 (21.8%)	13 (36.1%)	14 (23%)	11 (30.6%)	6 (21.4%)	
	Stage	Local	210 (79.5%)	30 (88.2%)	44 (75.9%)	26 (76.5%)	21 (80.8%)	0.68
		Regional or Distant	54 (20.5%)	4 (11.8%)	14 (24.1%)	8 (23.5%)	5 (19.2%)	
	Grade	1–2	200 (88.5%)	18 (66.7%)	47 (94%)	18 (78.3%)	18 (81.8%)	< 0.01
		3–4	26 (11.5%)	**9** **(33.3%)**	3 (6%)	5 (21.7%)	4 (18.2%)	
50 + years	Histology	Endometrioid	1071 (73.9%)	130 (52%)	189 (73.5%)	74 (71.2%)	51 (69.9%)	< 0.01
		Non-endometrioid	378 (26.1%)	120 **(48.0%)**	68 (26.5%)	30 (28.8%)	22 (30.1%)	
	Stage	Local	1045 (75.5%)	159 (67.7%)	167 (67.3%)	73 (76.8%)	52 (71.2%)	0.01
		Regional or Distant	340 (24.5%)	**76** **(32.3%)**	**81** **(32.7%)**	22 (23.2%)	21 (28.8%)	
	Grade	1–2	925 (82%)	100 (58.8%)	154 (73.7%)	52 (68.4%)	37 (67.3%)	< 0.01
		3–4	203 (18%)	**70** **(41.2%)**	55 (26.3%)	24 (31.6%)	18 (32.7%)	

Among low grade EEC cases, a lower proportion of Black patients had regional/distant stage disease (all ages, 5.8% versus ≥ 15.2%, compared to others P = 0.01) (Table 3). A smaller percentage of Black and Hispanic patients received adjuvant treatment (all ages, ≤ 13.1% versus 22.5–32.1% in other groups, P = 0.02). Similar patterns were observed among older (age 50 + years) cases. Differences by stage and receipt of adjuvant therapy across racial and ethnic groups were not observed among younger EEC cases.

**Table 3:** Patient tumor characteristics and adjuvant treatment by racial and ethnic group and age among patients with low grade (G1 and G2) endometrioid endometrial cancer

Age at diagnosis	Tumor/ treatment variable	Values	Non-Hispanic White	Black	Asian	Pacific Islander	Hispanic	p.value
All	Stage	Local	741 (82.2%)	81 (94.2%)	134 (77.5%)	41 (82.0%)	39 (84.8%)	0.01
		Regional or Distant	161 (17.8%)	5 (5.8%)	39 (22.5%)	9 (18.0%)	7 (15.2%)	
	Adjuvant therapy^a^	No	700 (77.5%)	73 (86.9%)	131 (75.3%)	36 (67.9%)	41 (89.1%)	0.02
		Yes	203 (22.5%)	11 (13.1%)	43 (24.7%)	17 (32.1%)	5 (10.9%)	
< 50 years	Stage	Local	130 (83.3%)	11 (91.7%)	30 (78.9%)	10 (76.9%)	13 (86.7%)	0.83
		Regional or Distant	26 (16.7%)	1 (8.3%)	8 (21.1%)	3 (23.1%)	2 (13.3%)	
	Adjuvant therapy^a^	No	127 (79.9%)	7 (58.3%)	27 (69.2%)	9 (64.3%)	14 (93.3%)	0.08
		Yes	32 (20.1%)	5 (41.7%)	12 (30.8%)	5 (35.7%)	1 (6.7%)	
50 + years	Stage	Local	611 (81.9%)	70 (94.6%)	104 (77.0%)	31 (83.8%)	26 (83.9%)	0.02
		Regional or Distant	135 (18.1%)	4 (5.4%)	31 (23.0%)	6 (16.2%)	5 (16.1%)	
	Adjuvant therapy^a^	No	573 (77.0%)	66 (91.7%)	104 (77.0%)	27 (69.2%)	27 (87.1%)	0.01
		Yes	171 (23.0%)	6 (8.3%)	31 (23.0%)	12 (30.8%)	4 (12.9%)	

^a^Adjuvant therapy was defined as receipt of radiotherapy only, chemotherapy only or both

In patients with localized disease, a higher percentage of Black patients had non-EEC histology (all ages, 35.4% versus ≤ 21.7% in other groups, P < 0.01) (Table 4). Black, Pacific Islander and Hispanic patients presented more often with high grade tumors (all ages, ≥ 19.6% compared to ≤ 11.8% in White and Asian patients, P < 0.01). A higher proportion of Black and Pacific Islander patients received adjuvant therapy (all ages, ≥ 25.7% versus ≤ 21.5% in others, P = 0.02). In patients > 50 years of age, these patterns remained with the exception that the receipt of adjuvant therapy was higher only in Pacific Islander patients (34.2%) versus other groups (≤ 24.2%, P = 0.02). In patients < 50 years of age, only differences by grade were observed across racial and ethnic groups (29.2% of Black patients had grade 3–4 disease versus ≤ 13.3% in others, P = 0.02).

**Table 4:** Endometrial cancer patient tumor characteristics and adjuvant treatment by racial and ethnic group and age among patients with localized disease

Age at diagnosis	Tumor/ treatment variable	Values	Non-Hispanic White	Black	Asian	Pacific Islander	Hispanic	p.value
All	Histology	Endometrioid	983 (78.3%)	122 (64.6%)	169 (80.1%)	78 (78.8%)	58 (79.5%)	< 0.01
		Non-endometrioid	272 (21.7%)	67 (35.4%)	42 (19.9%)	21 (21.2%)	15 (20.5%)	
	Grade	1–2	896 (88.2%)	104 (74.8%)	149 (88.7%)	52 (76.5%)	45 (80.4%)	< 0.01
		3–4	120 (11.8%)	35 (25.2%)	19 (11.3%)	16 (23.5%)	11 (19.6%)	
	Adjuvant therapy^a^	No	1011 (82.2%)	133 (74.3%)	164 (78.5%)	70 (71.4%)	56 (78.9%)	0.02
		Yes	219 (17.8%)	46 (25.7%)	45 (21.5%)	28 (28.6%)	15 (21.1%)	
< 50 years	Histology	Endometrioid	168 (80%)	21 (70%)	35 (79.5%)	20 (76.9%)	18 (85.7%)	0.69
		Non-endometrioid	42 (20%)	9 (30%)	9 (20.5%)	6 (23.1%)	3 (14.3%)	
	Grade	1–2	163 (92.1%)	17 (70.8%)	33 (97.1%)	13 (86.7%)	14 (87.5%)	0.015
		3–4	14 (7.91%)	7 (29.2%)	1 (2.94%)	2 (13.3%)	2 (12.5%)	
	Adjuvant therapy^a^	No	177 (87.2%)	17 (65.4%)	35 (81.4%)	22 (88%)	17 (85%)	0.08
		Yes	26 (12.8%)	9 (34.6%)	8 (18.6%)	3 (12%)	3 (15%)	
50 + years	Histology	Endometrioid	815 (78%)	101 (63.5%)	134 (80.2%)	58 (79.5%)	40 (76.9%)	< 0.01
		Non-endometrioid	230 (22%)	58 (36.5%)	33 (19.8%)	15 (20.5%)	12 (23.1%)	
	Grade	1–2	733 (87.4%)	87 (75.7%)	116 (86.6%)	39 (73.6%)	31 (77.5%)	< 0.01
		3–4	106 (12.6%)	28 (24.3%)	18 (13.4%)	14 (26.4%)	9 (22.5%)	
	Adjuvant therapy^a^	No	834 (81.2%)	116 (75.8%)	129 (77.7%)	48 (65.8%)	39 (76.5%)	0.02
		Yes	193 (18.8%)	37 (24.2%)	37 (22.3%)	25 (34.2%)	12 (23.5%)	

^a^Adjuvant therapy was defined as receipt of radiotherapy only, chemotherapy only or both

In patients with regional/distant disease, a higher proportion of Black patients had non-EEC and high-grade tumors than other groups (70% versus ≤ 50%; 79.2% versus ≤ 55%, respectively, P < 0.01 for both) (Table 5). These differences retained statistical significance in patients ages 50 + years (P < 0.01 for both) but not in younger patients. Pacific Islander patients received adjuvant therapy more often than other groups (96.7% versus ≤ 82.8%, P < 0.01). This pattern remained in patients 50 years and older but not in younger patients.

**Table 5:** Endometrial cancer patient tumor characteristics and adjuvant treatment by racial and ethnic group and age among patients with regional/distant disease

Age at diagnosis	Tumor/ treatment variable	Values	Non-Hispanic White	Black	Asian	Pacific Islander	Hispanic	p.value
All	Histology	Endometrioid	261 (66.2%)	24 (30%)	59 (62.1%)	15 (50%)	13 (50%)	< 0.01
		Non-endometrioid	133 (33.8%)	56 (70%)	36 (37.9%)	15 (50%)	13 (50%)	
	Grade	1–2	200 (65.6%)	11 (20.8%)	45 (53.6%)	12 (54.5%)	9 (45%)	< 0.01
		3–4	105 (34.4%)	42 (79.2%)	39 (46.4%)	10 (45.5%)	11 (55%)	
	Adjuvant therapy^a^	No	108 (28.1%)	19 (25.7%)	15 (17.2%)	1 (3.33%)	9 (36%)	< 0.01
		Yes	276 (71.9%)	55 (74.3%)	72 (82.8%)	29 (96.7%)	16 (64%)	
< 50 years	Histology	Endometrioid	41 (75.9%)	1 (25%)	10 (71.4%)	4 (50%)	2 (40%)	0.07
		Non-endometrioid	13 (24.1%)	3 (75%)	4 (28.6%)	4 (50%)	3 (60%)	
	Grade	1–2	31 (73.8%)	1 (33.3%)	11 (84.6%)	4 (66.7%)	3 (60%)	0.38
		3–4	11 (26.2%)	2 (66.7%)	2 (15.4%)	2 (33.3%)	2 (40%)	
	Adjuvant therapy^a^	No	12 (22.6%)	1 (25%)	1 (7.69%)	0 (0%)	1 (20%)	0.42
		Yes	41 (77.4%)	3 (75%)	12 (92.3%)	8 (100%)	4 (80%)	
50 + years	Histology	Endometrioid	220 (64.7%)	23 (30.3%)	49 (60.5%)	11 (50%)	11 (52.4%)	< 0.01
		Non-endometrioid	120 (35.3%)	53 (69.7%)	32 (39.5%)	11 (50%)	10 (47.6%)	
	Grade	1–2	169 (64.3%)	10 (20%)	34 (47.9%)	8 (50%)	6 (40%)	< 0.01
		3–4	94 (35.7%)	40 (80%)	37 (52.1%)	8 (50%)	9 (60%)	
	Adjuvant therapy^a^	No	96 (29%)	18 (25.7%)	14 (18.9%)	1 (4.55%)	8 (40%)	0.02
		Yes	235 (71%)	52 (74.3%)	60 (81.1%)	21 (95.5%)	12 (60%)	

^a^Adjuvant therapy was defined as receipt of radiotherapy only, chemotherapy only or both

In the present study there was a mean follow-up of 6.7 years (SD = 4.6) between the date of endometrial cancer diagnosis and the date of death or censoring. Risk of all cause mortality was compared across racial and ethnic groups after accounting for age at diagnosis, receipt of adjuvant therapy, histology and stage. We observed that Black patients had a higher mortality risk (Black patients with any type of endometrial cancer compared with White cases, HR 1.43, 95% CI, 1.13–1.83) (Table 6). Notably, this pattern persisted among cases with low grade EEC (Black compared with White cases, HR 2.26, 95% CI, 1.41–3.64) and for patients who presented with regional/distant disease (Black compared with White cases, HR 1.62, 95% CI, 1.15–2.28). In contrast, there were no differences in mortality risk for Asian, Pacific Islander or Hispanic patients when compared with White patients.

**Table 6:** Association between racial and ethnic groups and risk of all cause mortality among endometrial cancer patients overall and for selected case groups

Case groups	Value	Total N	Fatal Cases N (%)	HR (95% CI)^a^
Total endometrial cancer	Non-Hispanic White	1570	422 (26.9%)	1.00 (Ref)
	Black	250	89 (35.6%)	**1.43 (1.13–1.83)**
	Asian	285	60 (21.1%)	0.80 (0.61–1.06)
	Pacific Islander	120	17 (14.2%)	0.87 (0.53–1.43)
	Hispanic	93	23 (24.7%)	1.05 (0.67–1.64)
Low grade EEC^b^	Non-Hispanic White	869	194 (22.3%)	1.00 (Ref)
	Black	83	20 (24.1%)	**2.26 (1.41–3.64)**
	Asian	164	24 (14.6%)	0.73 (0.47–1.14)
	Pacific Islander	46	3 (6.5%)	0.53 (0.17–1.67)
	Hispanic	43	8 (18.6%)	0.93 (0.44–1.99)
Regional/distant disease	Non-Hispanic White	369	166 (45.0%)	1.00 (Ref)
	Black	71	53 (74.6%)	**1.62 (1.15–2.28)**
	Asian	89	43 (48.3%)	1.11 (0.79–1.57)
	Pacific Islander	29	7 (24.1%)	0.76 (0.35–1.67)
	Hispanic	24	11 (45.8%)	0.93 (0.47–1.87)

^a^ Adjusted for age at diagnosis (continuous); and receipt of the first course of adjuvant therapy [no adjuvant therapy (reference), radiation therapy, chemotherapy, radiation and chemotherapy, missing]; stratified by: histology [EEC, non-EEC]; and stage [localized, regional, distant]

^b^ Models included similar adjustment factors and strata variables as ^(a)^ except for histology

## Discussion

Prior publications have demonstrated increased rates of aggressive endometrial cancer histologies (non-EEC) in Black patients and associated worse survival [4, 12, 19]. Access to care has been postulated as a possible reason for worse survival in Black patients [23, 24] which prompted our inquiry into the ACTUR, a repository for data collected from patients treated in the Military Health System where access to care is universal. This study reaffirmed these trends in endometrial cancer including higher rates of non-EEC and high-grade histology in Black women [13, 14].

A novel finding in our study was that a higher percentage of Pacific Islander endometrial cancer patients received adjuvant therapy amongst all racial and ethnic groups and this persisted in most subgroup analyses. We did not however observe any consistent pattern of more aggressive endometrial tumor characteristics for Pacific Islander patients. The design of this study is better positioned to demonstrate differences in the receipt of adjuvant therapy because prior analyses have either not included Pacific Islander populations or have aggregated Pacific Islander with Asian patients [14, 25]. Including Asian and Pacific Islander patients together could mask aggressive endometrial cancer characteristics among the Pacific Islander population. In contrast, Asian women have been found to have similar rates of low grade tumors and EEC histology as White cases [26] and have improved overall and cancer-specific survival compared to other racial and ethnic groups [6] [26]. In the current study Asian women were more likely to have low grade tumors and EEC histology. We noted increased use of adjuvant treatment in Asian patients which is difficult to reconcile but, may be due to their increased presentation with more regional or distant stage disease. Regardless, it is of interest to further evaluate why a higher proportion of Pacific Islander patients received adjuvant treatment.

In the current study we observed that Black endometrial cancer patients had a higher risk of all cause mortality as compared with White patients. Inquiry into SEER and National Cancer Data Base (NCD) have reported similar increased mortality risk in Black women compared to White women across all ages [11, 27]. The SEER inquiry compared the patient’s age at diagnosis in relation to presentation with non-EEC tumors in Black and White women and they observed a trend toward increasing non-EEC histology in older women with a more pronounced increase for Black patients. In the review of ACTUR data presented here, there was concordance with the SEER data with no statistically significant difference in the proportion of cases with non-EEC in young women (< 50 years old) by race/ethnicity. However, there was a higher proportion of non-EEC histology in Black patients over the age of 50 compared to other racial and ethnic groups. There remain unknown drivers of aggressive disease that affect Black women variably.

Histologic typing of endometrial cancer has helped to dichotomize less aggressive (EEC) and more aggressive (non-EEC) disease however, molecular profiling has been tipped to add further stratification of aggressive disease. The Cancer Genome Atlas (TCGA) has been queried to investigate variance in molecular profiles of endometrial cancer and its association with outcome disparities. EEC is associated with microsatellite instability and mutations in *PTEN*, *PIK3CA*, *KRAS*, *POLE* and *CTNNBI* whereas non-EEC is associated with *TP53*, *ARID1A* and *HER2* overexpression for example [4]. Another study evaluated differences in endometrial cancer molecular profiles between ethnicities and found that Black or African American women were more likely to harbor *TP53* mutations (49%) versus White or Asian women who most often had mutations in *PTEN* (63% and 85%, respectively) [9]. As this data accumulates and molecular data continues to be used to drive treatment in oncology there is hope that a more targeted approach may be useful to address disparities in endometrial cancer.

Limitations of this study include small sample size when stratifying by racial and ethnic groups which may have limited our ability to identify meaningful differences in subgroups (age < 50 years in particular). Hispanic patients constituted a small subgroup in this study and when viewed in comparison to other groups represented a middling risk profile however, with increased power we could have better characterized these patients. In the ACTUR there were three methods of race and ethnicity reporting; two of the methods were based on self-report from the patient while the third method involved physician reports. Physician reported race and ethnicity is less reliable than self-reports from the patients. This could lead to misclassification of race and ethnicity and may attenuate risk estimates towards the null. Another limitation was the difficulty in stratifying on race and ethnicity as the groups included in this study cannot capture the diversity they represent (e.g., Pacific Islander: Native Hawaiian, Guamanian or Chamorro, Tongan, etc.). Furthermore, data were unavailable on body mass index, smoking status and on variables that are required to define the receipt of guideline concordant treatment [including detailed information on primary treatment (type of surgery, adjuvant treatment) and International Federation of Gynecology and Obstetrics (FIGO) staging]. Our study relied on registry data obtained by pathology reports generated at multiple sites which could lead to imprecise classification of histologic subtypes. It would therefore be useful to confirm these results in studies that have implemented a centralized pathology review. The primary strength of this study is that the Pacific Islander population was included and queried.

In summary, we observed more aggressive tumor features (non-endometrioid and high-grade histology) in Black patients and a higher proportion of Pacific Islander patients received adjuvant therapy. It has been hypothesized that differences in access to care may contribute in part to the observed racial and ethnic disparities in endometrial cancer mortality. Thus our goal was to determine whether there were differences in mortality risk across racial and ethnic groups in an equal access to care environment. Compared with White endometrial cancer patients, Black patients had a 1.4-fold higher risk of all cause death; these differences in mortality risk persisted in our US Military system study population where patients have equal access to healthcare. This supports the hypothesis that factors other than access to care may contribute to the observed racial and ethnic disparities in endometrial cancer mortality.

### Electronic supplementary material

Supplementary material

## Data Availability

This study used data from the data from the U.S. DoD ACTUR database which are not publically available due to privacy concerns. Data described in the manuscript, code book, and analytic code are available from the corresponding author upon reasonable request.
