# Supplementary material for: Racial and ethnic differences in tumor characteristics among endometrial cancer patients in an equal-access healthcare population
Source: Cancer Causes Control. 2023 Jul 12;34(11):1017–25. doi: 10.1007/s10552-023-01716-9 (PMC10533614; doi:10.1007/s10552-023-01716-9)
Supplement: Supplementary file 1 — Supplementary material [file 10552_2023_1716_MOESM1_ESM.doc]

**Desmond *et al*.** ‘Racial/ethnic differences in tumor characteristics among endometrial cancer patients in an equal-access healthcare population’

**Supplementary tables**

| **Supplementary Table 1.** International Classification of Diseases for Oncology 2^nd^ or 3^rd^ revision (ICD-O2 and -3, respectively) topography and morphology codes used to define invasive epithelial endometrial cancer and histological subtypes in the U.S. Department of Defense's Automated Central Tumor  Registry. | | | | |
| --- | --- | --- | --- | --- |
|  | **ICD-O2/3 topography code** | **ICD-O2/3 morphology code** | |  |
|  |  | Included | Excluded |  |
| **Epithelial endometrial cancer** | C54.0 - 54.9  C55.9 | N/A | 8000, 8141, 8120, 8440, 8800, 8801, 8804, 8805, 8890, 8900, 8930, 8931, 8933, 8935, 8990, 9100, 9105, 9364, 8891, 8896 |  |
| **Epithelial endometrial cancer subtypes by histology** | |  |  | **Total N=2574** |
| Endometrioid (including adenocarcinoma with squamous cell differentiation) | | 8380 |  | 1803 |
|  |  | 8382 |  | 6 |
|  |  | 8383 |  | 5 |
|  |  | 8033 |  | 1 |
|  |  | 8262 |  | 1 |
|  |  | 8384 |  | 1 |
|  |  | 8560 |  | 18 |
|  |  | 8570 |  | 15 |
|  |  | 8071 |  | 1 |
| Adenocarcinoma not otherwise specified (NOS) | | 8140 |  | 311 |
| Serous | | 8441 |  | 96 |
|  |  | 8460 |  | 18 |
|  |  | 8461 |  | 15 |
| Mucinous | | 8480 |  | 35 |
|  |  | 8482 |  | 3 |
| Clear cell | | 8310 |  | 20 |
| Other specified endometrial cancer | | 8950 |  | 31 |
|  |  | 8951 |  | 4 |
|  |  | 8980 |  | 71 |
|  |  | 8010 |  | 36 |
|  |  | 8050 |  | 3 |
|  |  | 8070 |  | 8 |
|  |  | 8246 |  | 1 |
|  | | 8255 |  | 10 |
|  | | 8260 |  | 4 |
|  | | 8323 |  | 55 |
|  | | 8562 |  | 1 |
|  | | 8574 |  | 1 |
